# Clinical pattern, neuroimaging findings and outcome of Acute Disseminated Encephalomyelitis in children: A retrospective study

**DOI:** 10.12669/pjms.40.7.8015

**Published:** 2024-08

**Authors:** Khairunnisa Mukhtiar, Mohammad Raza, Sidra Ishaque, Quratulain Maha, Ayesha Noor

**Affiliations:** 1 Khairunnisa Mukhtiar, Assistant Professor, Department of Pediatrics & Child Health, Section of Pediatric Neurology, Aga Khan University Hospital, Karachi, Pakistan; 2 Mohammad Raza, Instructor, Department of Pediatrics & Child Health, Section of Pediatric Neurology, Aga Khan University Hospital, Karachi, Pakistan; 3 Sidra Ishaque, Assistant Professor, Department of Pediatrics & Child Health, Section of Pediatric Critical Care Medicine, Aga Khan University Hospital, Karachi, Pakistan; 4 Quratulain Maha, Intern, Department of Pediatrics & Child Health, Aga Khan University Hospital, Karachi, Pakistan; 5 Ayesha Noor Intern, Department of Pediatrics & Child Health, Aga Khan University Hospital, Karachi, Pakistan

**Keywords:** Acute disseminated encephalomyelitis, Clinical spectrum, Neuroimaging findings, immediate outcome, Long term complications

## Abstract

**Objective:**

To determine the clinical spectrum, neuroimaging findings, and outcome of Acute Disseminated Encephalomyelitis (ADEM) in children.

**Method:**

We conducted a descriptive cross sectional study of all children aged 6 months to 18 years, diagnosed with ADEM at Aga Khan University Hospital, Karachi from January 2018 till December 2022.

**Results:**

This retrospective study enrolled 30 cases of ADEM, with a mean age of 6.43 ± 4.079, including 13 males and 17 females. The average hospital stay was 7.29 ± 4.379 days. The most common clinical features were fever, headache, and altered consciousness, while motor deficit was observed in 15 (53.5%) patients. Abnormal cerebrospinal fluid was found in 14 (46.6%) patients. Brain MRI identified bilateral and multifocal lesions in 22 (78.6%) patients, with brainstem lesions detected in 7 (25%) patients. Treatment included IV methylprednisolone (22; 73%), IVIG (9; 30%), or both (6; 20%). Clinical improvement was observed in 25 (89.3%) patients, with residual weakness present in eight (26%) patients at discharge. There was one reported death. Long-term complications included motor deficits, seizures, poor scholastic performance, and behavioral issues.

**Conclusion:**

The clinical presentation of ADEM is variable, but the most common symptoms are fever, headache, and altered consciousness. Despite generally favorable outcome, long-term monitoring revealed that patients may experience motor deficits, seizures, cognitive impairment, and academic difficulties.

## INTRODUCTION

Acute disseminated encephalomyelitis (ADEM) is an acute immune-mediated demyelinating disorder of the central nervous system that typically occurs after a viral infection or recent vaccination.^1^,^2^ The International Pediatric Multiple Sclerosis Society Group (IPMSSG) updated consensus definitions for demyelinating disorders of childhood, including ADEM in 2013.^3^ Although ADEM can occur at any age, it is more commonly seen in children, with a median age of onset 5-8 years.^4^,^5^ The annual incidence of pediatric ADEM is estimated to be 0.07 to 0.9 per 100,000 children, with a slight male predominance.^4^,^5^ The geographical distribution of ADEM cases seems to be similar to that of multiple sclerosis cases, with disease prevalence increasing as distance from the equator increases.^6^ Studies have suggested seasonal increase in incidence during the winter and spring.^1^,^7^

Clinical presentation of ADEM is variable, with initial symptoms comprising of prodromal symptoms such as fever, malaise, headache, nausea, and vomiting lasting 3-4 days before progressing to encephalopathy.^1^,^4^-^6^ Encephalopathy can be characterized by irritability, sleepiness, confusion, obtundation, or coma.^6^ Neurological manifestations are determined by the site of the lesions and include unilateral or bilateral pyramidal signs, ataxia, acute hemiparesis, seizures, cranial nerve palsies, visual changes, speech impairment, and spinal cord involvement.^5^,^8^,^9^ Some rare cases of severe hemorrhagic necrosis leading to rapid progression and death have also been reported, secondary to brainstem involvement.^7^

Patients may also present with atypical symptoms like pathological yawning secondary to brainstem involvement.^10^ A rare case of the fatal triad of ADEM, seizures and myocarditis has also been reported.^11^ Neuroimaging through magnetic resonance imaging (MRI) is essential for ADEM diagnosis, with lesions observed as hyper intense areas on T2-weighted and fluid-attenuated inversion recovery (FLAIR) imaging.^1^,^4^,^5^,^7^,^8^ Although not a typical feature of ADEM, gadolinium enhancement has been reported in 30% cases.^4^,^8^ ADEM lesions commonly involve the subcortical and central white matter and cortical gray-white matter junction, thalami, basal ganglia, cerebellum, and brainstem. The spinal cord can also be involved with large lesions extending over multiple segments. Cerebrospinal fluid (CSF) examination in ADEM usually has inconclusive results and is usually done to exclude other causes, especially infections, and may show inflammatory changes consisting of elevated protein levels and lymphocytic pleocytosis, or results can also be within normal limits.^1^,^5^-^7^

High-dose corticosteroids are considered the mainstay of treatment and are usually administered intravenously, followed by an oral taper. Intravenous immunoglobulin (IVIG) treatment has also been used as a second-line therapy in patients who do not respond to steroids.^1^,^5^ ADEM generally has a favorable outcome. With the early initiation of treatment, improvement can be seen as rapidly as within hours, but complete recovery may take days to weeks or months, depending on the severity of the disease.^9^ Complete recovery is reported in 56% to 94% of patients.^6^ Residual deficits commonly include motor deficits, ataxia, hemiparesis, and blindness.^8^ Subtle long-term neuropsychological deficits affecting attention, executive functioning, verbal processing, and behavior have also been observed.^8^ Fortunately, mortality during an acute episode is as low as 1-3%.^4^ In Pakistan there is a paucity of research done on clinical spectrum of ADEM in children, MRI brain findings and immediate and long-term outcome. This study presents comprehensive review of data regarding ADEM in children from Pakistan.

## METHOD

This was a descriptive cross sectional retrospective study and included all children diagnosed with ADEM at Aga Khan University Hospital in Karachi between January 2018 and December 2022. The diagnosis of ADEM was based on the IPMSSG 2013 revised criteria, and children aged 6 months to 18 years were eligible for inclusion.

### Ethical Approval

The study was approved by the Aga Khan University Ethics Review committee (ERC NO: 2020-3609-14606, Date: November 15, 2020).

Detailed information was gathered on several variables, such as demographic characteristics, duration of clinical symptoms, various neurological presentations (acute encephalopathy, seizures, headache, ataxia, weakness), cerebrospinal fluid findings, brain and spinal cord MRI findings, treatment administered, length of stay, need for mechanical ventilation, immediate outcome, and long-term neurological complications. All the information collected was used for a descriptive analysis. The immediate outcome was evaluated using the Modified Rankin Scale (MRS) score at discharge, with scores ranging from 0 to 5.

0. No symptoms.

1. No significant disability: Able to carry out all usual activities, despite some symptoms.
2. Slight disability: Able to look after own affairs without assistance, but unable to carry out all previous activities.
3. Moderate disability: Requires some help, but able to walk unassisted.
4. Moderately severe disability: Unable to attend to own bodily needs without assistance, and unable to walk unassisted.
5. Severe disability: Requires constant nursing care and attention, bedridden, incontinent

The long-term neurological outcome was assessed clinically through a chart review of the patient’s follow-up clinic visits at 6 months after discharge. Patients who were lost to follow-up were contacted via phone call to collect information. Data was analyzed using SPSS version 20.0. The results of the study were expressed as the mean, standard deviation, and range for continuous variables and as percentages for discrete variables.

## RESULTS

This study included 30 patients who were diagnosed with ADEM. The gender distribution was 43.3% male and 56.7% female, resulting in a male to female ratio of 0.76:1. The age range was 2 to 17 years with an average age of 6.43 ± 4.079 years. The hospital stay lasted an average of 7.29 ± 4.379 days. The highest number of cases, 12 (40%), occurred in the spring season, followed by seven (23%) in the winter season, with the rest of the cases observed in autumn and summer.

The most common clinical presentation was fever with headache in 15 patients (50%), with encephalopathy in 22 patients (73.3%). Additionally, 15 patients (50%) had motor deficits, seven patients (23.3%) had seizures, and two patients (6.6%) had visual symptoms. The clinical features of all patients are summarized in Table-I.

**Table-I:** Clinical Features of ADEM patients.

Characteristics	Total (n=30)
***Systemic sign and symptoms***	
Fever	5 (50%)
Nausea and vomiting	5 (16.6%)
Headache	15 (50%)
Neck stiffness	2 (6.6%)
***Neurological signs and symptoms***	
Motor deficits	15 (50%)
Encephalopathy	22 (73.3%)
Sensory deficits	5 (16.6%)
Cranial neuropathy	2 (6.6%)
Seizures	7 (23.3%)
Aphasia	2 (6.6%)
Visual disturbances	2(6.6%)
Bowel and bladder involvement	3 (10%)

All patients underwent CSF analysis, and 18 (60%) of them had normal results. However, elevated CSF proteins were found in 6 (20%) of the patients, and 10 (33.3%) had leukocytosis. Additionally, all patients had an MRI brain, which revealed multiple foci of increased signal intensity in T2 and FLAIR images in the cerebral white matter. The distribution of the lesions was as follows: subcortical white matter involvement was observed in 14 cases (46%), subcortical gray matter involvement was seen in 12 cases (40%), and brainstem involvement was present in seven cases (23%). Among the eight patients who underwent spinal MRI, three cases (10%) showed abnormal signals in the cervical region, as shown in Table-II.

**Table-II:** CSF and MRI Findings of patients.

Variables	Total (n=30)
***CSF^a^ analysis***	
Normal CSF	18 (60%)
Leukocytosis	10 (33.3%)
Elevated proteins	6 (20%)
MRI^b^Brain Findings	
***Location of hyperintense lesions on T2/FLAIR images***	
Frontal lobe	15 (50%)
Parietal lobe	14 (46.6%)
Temporal lobe	8 (26.6%)
Occipital lobe	6 (20%)
Cortical gray matter	12 (40%)
Subcortical white matter	14 (46%)
Periventricular white matter	9 (30%)
Internal capsule	1 (3.3%)
Thalamus	4 (13.3%)
Basal ganglia	3 (10%)
Brainstem	7 (23.3%)
Cerebellum	2 (6.6%)
Corpus callosum (Splenium)	1 (3.3%)
Spinal MRI done	8 (26.6 %)
Normal Spinal MRI	5 (16.6%)
Abnormal Spinal MRI	3 (10%)

a= cerebrospinal fluid, b= magnetic resonance imaging.

After the diagnosis was confirmed, all patients received standardized treatment. Intravenous methylprednisolone at a dose of 30 mg/kg/day for five days was given to 22 patients (73%). These patients were then administered prednisolone at a dose of 1-2 mg/kg/day for one month and the dosage was tapered off over three months. Intravenous immunoglobulin at a dose of 1 gram/kg/dose for two days was given to nine patients (30%). In cases where pulse methylprednisolone therapy was ineffective (no response after five days), IVIG was administered, and six patients (20%) received both treatments.

The mean length of hospitalization for the patients was 7.29 ± 4.37 days. Half of the patients 15 (50%) were admitted to the Pediatric Intensive Care Unit (PICU), and out of these patients, 10 (70%) required mechanical ventilation (MV) who had very low GCS <8 or brainstem lesions on MRI. The median duration of mechanical ventilation was 3.25 ± 1.73 days. The median period for detecting clinical improvement was five days, with a range of one to 12 days. Out of the total 30 patients, 96% (29) survived, while one patient passed away due to associated sepsis, as shown in Table-III.

**Table-III:** Management details and outcome of patients.

Variables	Total (n=30)
***Treatment given***	
High-dose steroids^a^	22 (73.3%)
IVIG^b^ - 2 doses	9 (30%)
Combined steroids & IVIG	6 (20%)
***Immediate Outcome***	
Survived	29 (96.6%)
Expired	1 (3.3%)
***MRS^c^score at the time of discharge***	
MRS score 2	17 (56.6%)
MRS score 3	7 (23%)
MRS score 4	3 (10 %)
MRS Score 5	2 (6.6%)
***Long term neurological complications (N=22)***	
No neurological deficit	15 (68%)
Residual motor deficit	4 (18%)
Seizures	4 (18%)
Poor Scholastic performance	6 (27%)
Cognitive impairment	4 (20%)
Behavioral issues	1(4.5%)

a= methylprednisolone pulse, b= intravenous immunoglobulin, c= modified rank in scale score.

Out of the total 29 patients who survived, 17 patients (56.6%) had an MRS score of 2, while 12 patients (43.3%) had a score ranging from 3 to 5, as indicated in Table-III. The study found that patients who required mechanical ventilation during their stay in the pediatric intensive care unit (PICU) and had a longer hospitalization period tended to have higher MRS scores at the time of discharge.

The long-term neurological outcome was assessed in 22 patients, but we did not study the risk factors associated with poor long term outcome. Of these patients, 15 (68%) had no signs of neurological deficits, while 4 (18%) experienced motor deficits, and four (18%) had seizures that necessitated the use of anti-epileptic medications. Additionally, six patients (27%) had poor scholastic performance, and one patient exhibited behavioral issues. It is worth noting that none of the patients developed multiple sclerosis during the follow-up period of six months.

## DISCUSSION

This study depicts the clinical and laboratory manifestations, and outcome of ADEM in children. We found that the encephalopathy was the most common presenting symptom of ADEM, while lymphocytic pleocytosis in CSF and subcortical white matter involvement on MRI were the other common findings. Few studies have been done from our country previously on ADEM in children.^12^,^13^ Zahra et al, reported a case of multiphasic ADEM in a four years old boy, who has two similar episodes of encephalopathy three months apart, and responded to steroids and IVIG.^12^ Rehman et al, studied functional outcome of ADEM in 15 children at Children hospital, Lahore. They found encephalopathy with subcortical white matter involvement as the common presenting features as in our study.^13^

ADEM is a monophasic inflammatory demyelinating disorder of the central nervous system. It occurs at any age but it is more frequent in childhood.^14^ The age range of patients in our study population was between 2 to 17 years, and the mean age was 7.29 ± 0.79 years. Our study findings are consistent with other studies that have shown a mean age of presentation below ten years.^15^,^16^

To establish a diagnosis of ADEM, the IPMSSG requires a presentation of multiple symptoms along with encephalopathy. Rehman et al, reported encephalopathy in 100% of patients with ADEM, as it was seen in 73% of patients in our study.^13^ The clinical presentation is characterized by a prodromal phase followed by the encephalitic phase, which typically starts within two days to four weeks and is marked by a decline in consciousness.^17^,^18^ The most common presentations of ADEM in our patients were encephalopathy and convulsions. Common systemic symptoms included fever, headache, nausea, and vomiting.

ADEM patients may exhibit a variety of neurological abnormalities including ataxia, hemiparesis, optic neuritis, and cranial nerve palsies. Long tract signs (unilateral or bilateral) are present in 85% of cases, while acute hemiparesis and ataxia are observed in 76% and 59% of cases, respectively. Cranial nerve palsies are found in 23-50% of cases, and seizures occur in 13-35% of pediatric cases. Optic neuritis, which is suggestive of the disease, is present in only 7-23% of cases.^15^ In our series, motor deficits were commonly seen in 50% patients, followed by sensory deficits 16%, cranial neuropathy 6%, visual disturbances 6%, and bladder and bowel involvement was seen in 10% patients.^17^,^18^

ADEM laboratory tests typically exhibit non-specific results with signs of inflammation. According to Erol et al.’s study, five out of 15 patients showed CSF lymphocytic pleocytosis, four of 15 patients had mild CSF protein elevation, and only one patient exhibited oligoclonal bands in the CSF.^19^ Tenembaum et al^20^ did not find any patient with oligoclonal bands in a series of 84 children with 54 CSF samples analyzed. In our patients, all underwent CSF analysis, and 60% of the results were normal. Pleocytosis was detected in 10 cases (33.3%), and six cases (20%) had a high protein concentration in the CSF. Our CSF results were consistent with other studies, but we did not check for oligoclonal bands.^21^,^22^

All patients in our study underwent MRI brain, revealing multiple foci of increased signal intensity in T2 and FLAIR images in the cerebral white matter. The sub-cortical and periventricular areas were the most affected, with involvement in 46% and 30% of patients, respectively. Cortical grey matter was involved in 40% of patients. These findings align with those of other studies.^16^,^23^ Basal ganglia involvement is common in ADEM, with thalamic lesions being particularly indicative of the disease, present in 13% of our cases. Corpus callosal lesions, although typically considered characteristic of multiple sclerosis, were present in only one of our patients, despite being reported in 13.4−29% of ADEM cases in previous literature.^24^

Rehman et al, reported good outcome (MRS score 2 or less) noticed in 80% patients at the time of discharge.^13^ Compared to this, MRS score was 2 in 56.6% of our patients, and a score of 3- 5 was seen in 43.3% patients. Similar findings were also observed by Iype M et al.^25^ Another study published by Shilo et al^26^ showed that high MRS scores at onset were associated with a poorer long-term outcome in pediatric ADEM patients, including a higher risk of developing MS. These studies suggest that MRS scores can serve as a useful prognostic tool in pediatric ADEM patients, aiding in long-term management and rehabilitation.

A study by Iype M et al, found that pediatric ADEM patients experienced long term neurological sequelae, including residual neurological deficits, language and memory issues, seizures, behavioral issues, poor scholastic performance in their large cohort with cognitive impairment and poor learning skills being the most common.^27^ In our study we were able to assess 22 out of 30 patients for the long term neurological complications. Among them, 15 (68%) had no neurological deficit, while the remaining seven patients reported some neurological complications such as motor deficits, seizures, poor scholastic performance, cognitive impairment, and behavioral issues. None of the patients in our cohort developed multiple sclerosis during the follow-up period. To our knowledge this is the only study from Pakistan in which we assessed ADEM for long-term neurological complications.

Our study adds significantly to the medical literature. It highlights the seasonal variation of ADEM, with the predilection for spring season. Brainstem involvement on MRI brain can be present as seen in 23% cases. Moreover, we found that median period for detecting clinical improvement was five days and patients who required mechanical ventilation during their stay in PICU had higher MRS scores at the time of discharge. Hence the need of mechanical ventilation indicates the disease severity and the risk of long term sequelae.

### Limitations

Firstly, it was retrospective, which may introduce biases inherent in this type of study. Secondly, our sample size was relatively small, which may restrict the generalizability of our findings. Lastly, we were unable to conduct long-term follow-up for all patients, which hampered our ability to monitor potential long-term consequences and the likelihood of developing multiple sclerosis

## CONCLUSION

The clinical presentation of acute disseminated encephalomyelitis is diverse, with fever, headache, and altered consciousness being the most common symptoms. Despite the variable clinical course and severity of the disease, the majority of patients showed favorable clinical outcomes. However, a minority of patients developed long-term neurological deficits, such as motor deficits, seizures, poor scholastic performance, cognitive impairment, behavioral issues, which highlights the need for long-term follow-up of these patients for the identification and early intervention for improvement in the functional outcome in children.

### Authors’ Contribution:

**KM, MR** and **SI:** Designed the study and prepared the initial draft.

**QM, AN** and **DS:** Collected the data and wrote discussion.

**KM, MR, SI** and **QM:** Devised methodology and analyzed the data.

**KM, MR** and **SI:** Wrote and revised the final manuscript.

**KM:** He is also responsible the accuracy and integrity of this manuscript.
